# Undernutrition and Its Predictors Among Pregnant Women in Semipastoral Communities of Southwest Ethiopia

**DOI:** 10.1155/jnme/8530014

**Published:** 2025-07-17

**Authors:** Getaneh Workineh, Abyot Asres

**Affiliations:** ^1^School of Public Health, College of Medicine and Health Sciences, Mizan Tepi University, Mizan Teferi, Ethiopia; ^2^Bachuma Hospital, West Omo Zone, Southwest Ethiopia Region, Bachuma, Ethiopia

**Keywords:** antenatal care, Ethiopia, hospital, pregnant, undernutrition

## Abstract

Maternal undernutrition poses significant risks to maternal, fetal, and child health, leading to long-term and irreversible consequences such as growth failure. This study evaluated the prevalence and predictors of undernutrition among pregnant women attending antenatal care at Bachuma Primary Hospital in Southwest Ethiopia. Using an institution-based cross-sectional design, 346 randomly selected pregnant women were assessed for nutritional status based on mid-upper arm circumference (MUAC), with values below 23 cm indicating undernutrition. Data analysis revealed that 31.5% of pregnant women were undernourished (95% CI: 26.6%–36.7%). Predictors of undernutrition included low dietary diversity scores (< 5) (AOR = 1.88; 95% CI (1.06–3.33)), consuming fewer than three meals daily (AOR = 2.87; 95% CI (1.29–6.36)), high parity (gravida six or more) (AOR = 4.98; 95% CI (1.06–23.4)), and intestinal parasitic infections (AOR = 2.86; 95% CI (1.58–5.18)). The study highlights the urgent need for interventions such as enhanced dietary counseling, improved maternal nutrition practices, and the screening and treatment of parasitic infections to mitigate undernutrition in this population.

**Summary**

• The magnitude of undernutrition in pregnant women in semipastoral areas is higher than that in agrarian areas.
• Predictors for undernutrition in pregnant women are related to maternal dietary practices, obstetric factors, and concomitant intestinal parasite infection.
• Predictors for undernutrition in pregnant women in semipastoral communities are similar to those identified in agrarian areas.

## 1. Introduction

Maternal nutrition before, during, and after birth is a key for fetal growth, birth weight, and infant well-being. In contrast, poor maternal nutrition often leads to long-term, irreversible and detrimental consequences to the fetus that result in intergenerational growth failure [1]. The most damaging effect of undernutrition occurs during pregnancy and in the first 2 years of life [2]. Thus, optimal maternal nutrition during the “first 1000 days” window from conception through the first six months of life is critically important to reduce adverse health outcomes for women and their infants [3–5].

Globally, more than 3.5 million mothers and children under 5 years die each year and millions more are disabled due to undernutrition occurring before, during, and after birth [6]. Despite adequate food for everybody on earth, maternal undernutrition is still common and associated with millions of deaths globally every year [7].

Maternal undernutrition is prevalent in low- and middle-income countries, resulting in substantial mortality and overall disease burden [6]. Prevalence of maternal undernutrition is as high as 35% in some countries of Southeast Asia, South America, and African regions [8]. A systematic review and meta-analysis of studies in Africa and Ethiopia showed prevalence of undernutrition in pregnant women of 23.5% in Africa and 29.07% in Ethiopia [9]. Empirical studies in different parts of Ethiopia also reported diverse levels of undernutrition in pregnant women [10–14], ranging from 14.4% [13] in Gondar town, Northern Ethiopia, to 52.6% [14] in Kacha Bira district of Central Ethiopia.

In pastoralist communities, nutritional status is heavily influenced by seasonal changes, with malnutrition becoming more severe during droughts, exacerbated by climate change. Common micronutrient deficiencies include iron, folic acid, and niacin. Pregnant women frequently adhere to dietary restrictions and food taboos, believing these practices will ease childbirth. Notably, dietary taboos are prevalent among 67.4% of pastoral communities in Eastern Ethiopia [15].

Pastoralist communities not only face chronic undernutrition but also significant health challenges from diseases like malaria, respiratory infections, and diarrhea. Limited access to healthcare and safe drinking water intensifies these issues, with water-borne diseases being especially common. Additionally, the shift to sedentarization has introduced problems such as inadequate housing and restricted access to clean water, further worsening their nutritional status [16].

In rural Chad, malnutrition among women in pastoralist areas reached a prevalence of 36%, with cases surging toward the end of the wet season. Additionally, 75% of women were reported to have intestinal parasitic infections, while 53% of pregnant women suffered from anemia [17].

Women in pastoral communities face culturally defined roles shaped by social, cultural, and economic factors. A study in a Kenyan pastoral community reported maternal undernutrition as a key factor associated with increased risks of fetal or perinatal death. Contributing factors include severe morbidity episodes, shorter pregnancy intervals, lower third-trimester weight, higher third-trimester skinfold measurements with limited reduction, and elevated activity levels late in pregnancy [18].

Studies exploring predictors of undernutrition in pregnant women revealed various attributes related to the pregnancy, household (HH), and residence. Accordingly, pregnant women older than 30 years [19], single or divorced or separated women [20], low educational status [21], dietary diversity score less than five [10, 12], food insecurity [12], meal frequency less than three times per day [22], multigravida [23], being at third trimester [24], being anemic [25], having family size greater than six [26], infected by intestinal parasite (IP) [27], and polygamous [24] had higher odds of undernutrition.

Studies conducted in different parts of the Ethiopia and elsewhere used different cutoffs of mid-upper circumference to determine undernutrition among the pregnant women despite nationally recommended value of 23 [28]. In addition, the studies are limited to agrarian agricultural areas of the country. As a result, evidence on undernutrition in pregnant women in pastoral and semipastoral areas is scanty. The pastoral and semipastoral communities in the southwest border of Ethiopia depend on animal husbandry supplemented with small-scale agriculture, wild plants, and hunting wild animals. The contents and diversities of the food items do have impact on nutritional status of the community. Hence, this study was aimed to assess magnitude and predictors of undernutrition in pregnant women in pastoral and semipastoral communities with diverse feeding practices.

## 2. Methods

### 2.1. Study Setting

The study was conducted at Bachuma Primary Hospital in semipastoral communities of Southwest Ethiopia. The catchment area of the hospital contains 64 administrative kebeles where more than 90% of population lives in rural areas. The main economic activity of the community around the hospital is small-scale subsistence agriculture supplemented by hunting of wild edible animals and gathering of wild edible plants [29]. Among the total pregnant women in the district, 81.4% women underwent antenatal care (ANC) checkup at Bachuma Hospital in 2023. Iron folate supplementation, deworming, and nutritional counseling were the services provided during ANC follow-up [30].

### 2.2. Study Design and Sampling

An institution-based cross-sectional study was conducted from June 15 to July 30, 2023, among pregnant women attending ANC visit at Bachuma Primary Hospital. Sample size was calculated by using Epi Info StatCalc Version 7 software by considering 29% proportion of undernutrition [31], 95% confidence level, 5% margin of error, and 10% nonresponse rate that computed and gave sample size of 348 pregnant women attending ANC. A systematic random sampling technique was employed to select the study subjects. The samplinginterval was determined based onthe preceding three months ANC attendance reportthat was reported as 804. Thus, every other pregnant woman visiting ANC clinic was consecutively enrolled to the study until required sample size was reached. During the selection, pregnancy confirmed via urine test and obstetric ultrasound was included, and those with severe disease, chronic disease, mental problems, and/or unable to communicate during data collection were excluded from the study.

### 2.3. Study Variables and Measurement

The dependent variable is undernutrition measured as yes or no, and gynecological/obstetric and maternal factors (parity, gravidity, gestational week, and history of abortion), maternal care and health-related factors (anemia, IP, previous modern family planning usage, iron supplementation intake in current pregnancy, and duration of supplemental iron intake), dietary intake adequacy (minim dietary diversity for women of reproductive age, meal frequency, and food security), and environmental factors (flooring type of the HH, water sources, and having toilet) were measured as explanatory variables.

A structured and pretested questionnaire was used to collect sociodemographic, nutrition-related, obstetric, health, and environmental factors. Pregnancy status and gestational age were extracted from the ANC service register since all pregnant women undergo obstetric ultrasound exam routinely at their first visit. The Minimum Dietary Diversity for Women (MDD-W) questionnaire was adapted from the Food and Agriculture Organization (FAO), and it was calculated from a single 24 h recall prior to data collection [32, 33]. All foods and beverages consumed in a day before study were grouped into 10 categories. Consuming a food item from any of the predefined food groups was assigned a score of 1 and if no food was taken a score of 0 was given by open recall method. Foods consumed by the study participant but not on predefined food list were recorded on a separate sheet and incorporated to the predefined list later by the enumerator. Accordingly, MDD-W up to 10 points was computed by adding the scores which were classified as inadequate when below five and adequate when at least 5 out of 10 predefined food groups were consumed. The 10 food groups are, namely, (1) grains, white roots and tubers, and plantains, (2) pulses (beans, peas, and lentils), (3) nuts and seeds, (4) dairy, (5) meat, poultry, and fish, (6) eggs, (7) dark green leafy vegetables, (8) other vitamin A–rich fruits and vegetables, (9) other vegetables, and (10) other fruits [32].

HH food security status was determined by using standard set of questions derived from Household Food Insecurity Access Scale (HFIAS) measurement guide [34]. The guide consists of nine occurrence questions that represent a generally increasing level of severity of food insecurity level with minimum score of 0 and maximum score of 27 HH food insecurity indicators. Finally, those respondents who scored below two out of 27 were categorized as food secured and those scored two and more (≥ 2) out of 27 on the questionnaire were categorized as food insecure.

There is no global agreement on the best anthropometric measurement to assess nutritional status of pregnant women. Mid-upper arm circumference (MUAC) is simpler to measure than other indicators and is not affected by pregnancy. Thus, several countries have established cutoffs for classifying malnutrition in women who are pregnant [35]. Current data suggest that the most conservative cutoff value of 23 cm appears adequate for Asian and African continents [36]. MUAC was measured halfway between the tip of the shoulder (olecranon process) and the tip of the elbow (acromion process) to the nearest 0.1 cm. An insertion type of adult MUAC tape that is nonelastic and nonstretchable was used to take the measurement. The measurement was taken at the mid-point on the relaxed left arm, without any clothing and with optimal tape tension (not too loose or not too tight) following the standard instructions and steps [37].

A fresh venous blood of about 20 mcl was taken from each participant following standard aseptic procedures. A precalibrated instrument was designed for measurement of hemoglobin concentration and labeled with identification number. The venous blood was drawn through micro cuvettes and inserted into HemoCue Hb analyzer and the result was recorded [38].

A stool sample was collected from each participant using stool specimen container with an applicator stick. Direct wet mount and formaldehyde-ether sedimentation methods were performed for efficacy of detecting IP. The World Health Organization (WHO) standard operating procedure for diagnosis of IP was used as an identification reference [39]. Laboratory investigation was carried out by two laboratory technologists.

### 2.4. Operational Definitions

Anemia was ascertained based on WHO criteria for anemia during pregnancy which describes that hemoglobin level below 11 g/dL during first and third trimester, and hemoglobin level below 10.5 g/dL during second trimester [40].

Undernutrition: MUAC less than 23 was considered as undernutrition [28].

MDD-W: Consuming of less than five food groups out of 10 predefined food groups [32].

Food secure: Respondents with HH scored less than two out of 27 by using HFIAS score [34].

Adequate access to improved source of water: Getting water from piped or protected ground water (wells and springs) source within 1 km or within 15 minute walking distance [41].

### 2.5. Data Processing

Each completed questionnaire was coded and entered into EpiData Version 4.6 and exported to SPSS Version 25 for analysis. Descriptive statistics and bivariable and multivariable binary logistic regression models were fitted to determine the association between dependent and independent variables. Variables with *p* value ≤ 0.25 by bivariable analysis were entered to multivariable analysis to control the effect of confounder. Hosmer–Lemeshow goodness of fit test statistics were used to test model fitness (*p* = 0.57). Variance inflation factor (VIF) was used to check multicollinearity among predictor variables (it was below 4).

## 3. Results

### 3.1. Sociodemographic Characteristics of Study Participants

A total of 348 pregnant women were planned to be studied and 346 completed, making the response rate 99.7%. Two-thirds of the respondents (66.8%) belong to ethnic Meinits. The median age of respondents was 25 years (interquartile range (IQR) = 23–29). Nearly half of the respondents (48.8%) were between age categories of 25–34 years. Majority of the respondents (273 (78.9%)) were rural dwellers, and 95.4% of were married of whom 50 (15.1%) were having polygyny (having at least one co-wife). More than half (194 (56.1%)) of the women had HH size of four and more (Table 1).

### 3.2. Dietary Intake Pattern and Environmental Conditions of Study Participants

As shown in Table 2, 210 (60.7%) of them had adequate micronutrient intake (MDD-W ≥ 5). The most commonly and frequently consumed food groups were foods made from grains, white roots and tubers, and plantains (91.6%) and dark green vegetables (90.8%) (Table 2).

### 3.3. Gynecological/Obstetric, Maternal Care, and Health-Related Factors

Two hundred and eleven (61%) women had gravidity between two and five, and 185 (53.5%) and 92 (26.6%) women were at their first trimester of pregnancy. One hundred eighty five (53.5%) of the respondents had been taking iron supplement during the current pregnancy of whom 31 (16.8%) had taken for more than three months. More than half (207 (59.8%)) of the study participants were infected by one or more type of IP. One-third (115 (33.2%)) of the pregnant women had previous history of abortion and 237 (68.5%) women had ever used family planning service (Table 3).

### 3.4. Magnitude and Factors Associated With Undernutrition

The mean MUAC (±SD) of the study participants was 23.2 (±0.9) with minimum and maximum values of 20.6 and 25.8, respectively. Accordingly, the overall magnitude of undernutrition among pregnant women attending ANC based on the MUAC below 23 was 31.5% (95% CI: 26.6%–36.7%).

### 3.5. Factors Associated With Maternal Undernutrition

The multivariable binary logistic regression analysis revealed that MDD-W ≤ 5, meal frequency below three per day, gravidity more than six, and IP infection were predictors of undernutrition. Pregnant women with low MDD-W below five had about twofold (AOR = 1.88: 95% CI (1.06–3.33)) higher odds of undernutrition. Similarly, pregnant women who were served less than three meals per day had about three times higher odds of undernutrition (AOR = 2.87; 95% CI (1.29–6.36)). Pregnant women who were gravida six and more had about five times (AOR = 4.98; 95% CI (1.06–23.4)) higher odds of undernutrition compared to those having below six gravida. Pregnant women who had intestinal parasitic infection were about three times more likely to be undernourished than those who had no IP infection (AOR = 2.86; 95% CI (1.58–5.18)) (Table 4).

## 4. Discussion

This study assessed the magnitude and associated factors of undernutrition among pregnant women attending ANC visit at Bachuma Primary Hospital. The finding revealed that the prevalence of undernutrition was 31.5% and minimum dietary diversity score less than five, meal frequency less than three per day, gravidity greater than six, and IP infection were significantly associated with undernutrition.

This proportion of undernutrition was consistent with studies in Mieso Health Center semipastoral area of West Hararghe, Eastern Ethiopia [42], which was 30.3%, 30.9% in pastoral community of Afar Region, Ethiopia [12], and 27% in Kenya [20]. The study was not in line with studies in Kacha Bira district of Kembata, Ethiopia, which was 52.6% [14], 43.1% in Konso district, Southern Ethiopia [11], 42.4 in Bench Sheko and Kaffa, Southwestern Ethiopia [33], and 21.8% in Silt'e Zone, Central Ethiopia [19]. The most probable reason for the variation could be the difference in cut points of MUAC measurements to determine undernutrition between studies. In addition, the difference may be due to geographical, sociodemographic, sociocultural, and dietary habit variation and seasonal differences during data collection.

Minimum dietary diversity (MDD-W) was one of the factors significantly associated with maternal undernutrition. The odds of undernutrition among pregnant women with low minimum dietary diversity were higher than women with high minimum dietary diversity. The finding was consistent with previous study findings at Nigist Eleni Mohammad Memorial Hospital, Central Ethiopia [22], Konso, Southern Ethiopia [11], Dessie, Northern Ethiopia [43], Kacha Birra district of Southern Ethiopia [14], Dire Dawa, Eastern Ethiopia [44], Gambella, Western Ethiopia [45], and Guji, Southeastern Ethiopia [21]. This might be because of inadequate intake of nutrients to meet the increased need of metabolic activities of women and their fetus.

Pregnant women who took meal less than three times per a day were more undernourished than women who took meal more frequently during pregnancy. Similar findings were observed in earlier studies in Arsi, Central Ethiopia [23], Nigist Eleni Mohammad Memorial Hospital [22], Kunama [46], and in Gumay district of Jimma, Southwestern Ethiopia [10]. The probable reason may be due to inadequate food available to share with families so that women may skip regular meals. In addition, women may be busy with livestock rearing and other HH chores and may be exhausted to eat their meals. This leads to decreased caloric supply for women and their fetus during pregnancy that results in fat depletion and subsequent undernourishment.

Pregnant women who become pregnant six times and more were five times more likely to be undernourished than their counterparts. The finding was consistent with the study conducted in Tena district of Arsi, Central Ethiopia [23]. This could be due to the fact that women had short time to recover from pregnancy related body changes thatdemandfor rebuild of energy and blood loss. In addition, women might be undernourished as they share nutrients to their infant during lactation and their fetus during pregnancy.

Intestinal parasitic infection was one of the predictor variables of undernutrition. The study finding was similar with previous study [47]. The reason behind this might be that IP infection causes undernutrition by decreasing appetite, inducing vomiting and diarrhea, preventing nutrient absorption, directly competing for nutrients, and inducing gastrointestinal bleeding. In addition, pastoral communities do lack access to safe water and sanitation services as they move from place to place for search of grass and water for their livestock.

Major limitation of the study was that it was an institution-based study that may not represent the general population. Additionally, the study was also prone to recall and social desirability bias for the data collection on dietary diversity and food security section for which the data collectors were trained to ask different indirect and probing questions to minimize these biases.

### 4.1. Significance and Policy Implication of the Study

Based on the findings of the study, here are the key policy implications:

1. Promote maternal nutrition programs: The high prevalence of undernutrition among pregnant women highlights the need for targeted maternal nutrition programs that improve dietary diversity and access to nutritious foods, especially in semipastoral communities.
2. Strengthen dietary counseling: Incorporating robust prenatal dietary counseling services within ANC programs can help address inadequate meal frequency and low dietary diversity.
3. Control and prevent parasitic infections: The association between intestinal parasitic infections and undernutrition underscores the need for routine screening and treatment of such infections during pregnancy through integrating deworming campaigns and hygiene education into maternal health services.
4. Address obstetric risk factors: Policies should encourage family planning and education to manage high-parity pregnancies (gravida six and above), which are linked to higher odds of undernutrition.
5. Improve healthcare accessibility: Strengthening healthcare infrastructure and outreach in underserved areas can ensure that pregnant women receive timely care, nutritional support, and treatment for infections.
6. Community engagement and awareness: Collaborating with community leaders and stakeholders to raise awareness about the importance of maternal nutrition and health facility services can foster cultural acceptance and enhance participation in health programs.

## 5. Conclusion and Recommendation

The study revealed that a significant proportion of pregnant women were undernourished in the study area for which maternal dietary practices, obstetric factors, and concomitant infections predicted higher odds of undernutrition. Thus, proper dietary counseling, provision of obstetric care, provision of safe water and sanitation, and screening and treatment of parasitic infections are required to avert the undernutrition. Furthermore, community-based follow-up studies starting from prepregnancy body mass index (BMI) level and changes during pregnancy along with biochemical tests are required to comprehensively understand the undernutrition in pregnant women.

## Figures and Tables

**Table 1:** Sociodemographic characteristics of study participants at Bachuma Primary Hospital, Southwest Ethiopia, June 15–July 30, 2023 (*n* = 346).

Variable	Category	Frequency	Percentage (%)
Age in years (*N* = 346)	15–24	147	42.5
	25–34	169	48.8
	35–49	30	8.7

Marital status (*N* = 346)	Married	331	95.7
	Divorced	4	1.2
	Widowed	9	2.6
	Single	2	0.6

Religion (*N* = 346)	Orthodox	57	16.5
	Muslim	9	2.6
	Protestant	276	79.8
	Catholic	2	0.6
	Others^1^	2	0.6

Ethnicity (*N* = 346)	Meinit	231	66.8
	Bench	37	10.7
	Kaffa	23	6.6
	Amhara	34	9.8
	Others^2^	21	6.1

Educational status of women(*N* = 346)	Illiterate	230	66.5
	Can read and write	92	26.6
	Above primary education	24	6.9

Head of household (*N* = 346)	Father	295	85.3
	Mother	47	13.6
	Others^3^	4	1.2
Occupation of household head (*N* = 346)	Farmer	271	78.3
	Merchant	36	10.4
	Others^4^	39	11.3

Educational status of household head (*N* = 346)	Illiterate	170	49.1
	Can read and write	142	41
	Above primary education	34	9.8

^1^Traditional believer.

^2^Dizi, Kembata, and Sidama.

^3^Uncle, sister, and brother.

^4^Government employees and daily laborer.

**Table 2:** Dietary intake adequacy and environmental conditions of pregnant women at Bachuma Primary Hospital, Southwest Ethiopia, from June 15 to July 30, 2023 (*N* = 346).

Variable	Category	Frequency	Percentage (%)
MDD-W^∗^	≥ 5	210	60.7
	< 5	136	39.3

Meal frequency	≥ 3 meal/day	304	87.9
	< 3 meal/day	42	12.1

Food security	Secure	246	71.1
	Insecure	100	28.9

Adequate access to improved source of water	Yes	123	35.5
	No	223	64.5

Owning toilet facility	Yes	193	55.8
	No	153	44.2

Material house floor	Cement	28	8.1
	Mud	318	91.9

^∗^MDD-W—Minimum Dietary Diversity for Women.

**Table 3:** Gynecological/obstetric, maternal care, and health-related factors of pregnant women in Bachuma Primary Hospital, Southwest Ethiopia, from June 15 to July 30, 2023.

Variable	Category	Frequency	Percent (%)
Gravidity (*N* = 346)	1	88	25.4
	2–5	211	61
	≥ 6	47	13.6

Parity (*N* = 346)	0	103	29.8
	1–3	210	60.7
	≥ 4	33	9.5

Gestational age in trimester (*N* = 346)	First	92	26.6
	Second	143	41.3
	Third	111	32.1

Age of last child in year (*N* = 233)	< 2	101	43.3
	≥ 2	132	56.7

Duration of iron intake (*N* = 185)	One month	66	35.7
	Two months	88	47.6
	≥ 3 months	31	16.8

Hemoglobin level (*N* = 346)	≥ 11 or (10.5 in 2^nd^ TM) gm/dL	239	69.1
	< 11 (10.5 in 2^nd^ TM^∗^) gm/dL	107	30.9

^∗^TM—trimester.

**Table 4:** Predictors of undernutrition in pregnant women at Bachuma Primary Hospital, Southwest Ethiopia, from June 15 to July 30, 2023.

Predictor variable	Category	Undernutrition		COR (95% CI)	AOR (95% CI)	*p* value
		Yes	No			
MDD-W (*N* = 346)^∗∗^	< 5	63	73	3.1 (1.92–4.92)	1.88 (1.06–3.33)	0.03^∗^
	≥ 5	46	164	1	1	

Meal frequency (*N* = 346)	< 3	26	16	4.33 (2.21–8.47)	2.87 (1.29–6.36)	0.01^∗^
	≥ 3	83	221	1	1	

Number of gravida (*N* = 346)	1	19	69	1	1	
	2–5	66	145	1.65 (0.92–2.97)	2.39 (0.63–8.97)	0.20
	≥ 6	24	23	3.79 (1.74–8.14)	4.98 (1.06–23.4)	0.04^∗^

Parity (*N* = 346)	0	24	79	1	1	
	1–3	72	138	1.72 (1.002–2.95)	0.75 (0.2–2.78)	0.66
	≥ 4	13	20	2.14 (0.93–4.93)	0.3 (0.06–1.64)	0.17

Family size (*N* = 346)	< 4	38	114	1	1	
	≥ 4	71	123	1.73 (1.08–2.77)	0.84 (0.42–1.72)	0.64

Supplemental iron intake (*N* = 346)	Yes	47	138	1	1	
	No	62	99	1.84 (1.16–2.91)	1.65 (0.96–2.85)	0.07

Intestinal parasite infection (*N* = 346)	No	23	116	1	1	
	Yes	86	121	3.59 (2.12–6.07)	2.86 (1.58–5.18)	0.001^∗^

Anemia (*N* = 346)	No	46	176	1	1	
	Yes	63	61	2.11 (1.31–3.40)	0.85 (0.46–1.56)	0.6

Household food security (*N* = 346)	Yes	63	181	1	1	
	No	44	56	2.19 (1.35–3.56)	1.55 (0.86–2.81)	0.15

^∗^ *p* value is significant at < 0.05.

^∗∗^ indicates significantly associated predictors.

## Data Availability

The data that support the findings of this study are available from the corresponding author upon reasonable request.

## Nomenclature

ANC: Antenatal care
AOR: Adjusted odds ratio
BMI: Body mass index
DDS: Dietary Diversity Score
HMIS: Health management information system
HFIAS: Household Food Insecurity Access Scale
IQR: Interquartile range
MCH: Maternal and child health
MDD-W: Minimum Dietary Diversity for Women
MUAC: Middle upper arm circumference
OR: Odds ratio
TM: Trimester
VIF: Variance inflation factor
WHO: World Health Organization
